# Genomic single nucleotide polymorphisms in the offspring of gastric cancer patients predispose to spasmolytic polypeptide-expressing metaplasia after *H. pylori* infection

**DOI:** 10.1186/s12929-015-0121-7

**Published:** 2015-02-21

**Authors:** Yu-Ching Tsai, Wei-Hsin Hsiao, Sheng-Hsiang Lin, Hsiao-Bai Yang, Hsiu-Chi Cheng, Wei-Lun Chang, Cheng-Chan Lu, Bor-Shyang Sheu

**Affiliations:** Departments of Institute of Clinical Medicine, National Cheng Kung University Hospital, College of Medicine, National Cheng Kung University, Tainan, Taiwan; Department of Internal Medicine, National Cheng Kung University Hospital, 138 Sheng Li Road, Tainan, 70428 Taiwan; Departments of Pathology, National Cheng Kung University Hospital, College of Medicine, National Cheng Kung University, Tainan, Taiwan; Department of Internal Medicine, Tainan Hospital, Department of Health, Executive Yuan, Tainan, Taiwan; Department of Pathology, Ton-Yen General Hospital, Hsin-Chu, Taiwan

**Keywords:** Gastric cancer, Cancer relatives, *H. pylori*, Corpus predominant gastritis index (CGI), Spasmolytic polypeptide-expressing metaplasia (SPEM), Single nucleotide polymorphisms, COX-2, IL-10, Integrin a5b1, COX-2, Trefoil factor 2

## Abstract

**Background:**

Gastric cancer exhibits familial clustering, and gastric cancer familial relatives (GCF) tend to present with corpus-predominant gastritis and precancerous lesions as SPEM or IM after *H. pylori* infection. The study determined whether the children of gastric cancer patients (GCA) had genomic single nucleotide polymorphisms (SNPs) predisposed to the gastric precancerous lesions as spasmolytic polypeptide-expressing metaplasia (SPEM) or intestinal metaplasia (IM).

**Results:**

There were 389 family relatives of 193 non-cardiac GCA and 173 duodenal ulcer patients (DU), received blood sampling for DNA collection. The differences of the risk alleles of SNPs in the *ITGA5*, *ITGB1*, *IL-10*, *COX-2*, *RUNX3*, and *TFF2* genes were compared between 195 children of GCA and 143 DU. The children of GCA had higher allele frequencies of *ITGA5*-1160 T-carrier (*P* = 0.006, OR[95% CI] = 2.2[1.2-4]), *ITGB1*-1949 A-carrier (*P *= 0.047; OR[95% CI] = 2.8[1.4-5.3]), *ITGB1* + 31804 C-carrier (*P *= 0.013; OR[95% CI] = 4.7[1.7-13.0]), *IL-10*-592 AA (*P *= 0.014; OR[95% CI] = 2.3[1.4-4.0]) and *COX-2-*1195 G-carrier (*P *= 0.019; OR[95% CI] = 1.7[0.9-3.2]) than DU. The combined genotype with *ITGA5*-1160/*ITGB1*-1949/*ITGB1* + 31804 as T/A/C carriers and *COX-*2-1195/*IL-10*-592 as G-carrier/AA was more prevalent in the children of GCA than in DU (*P* < 1×10^−4^), and predisposed with a 5.3-fold risk of getting SPEM in the *H. pylori*-infected children of GCA (*P *= 0.016). Such risk of getting SPEM increased to 112 folds, if combined with *RUNX3* + 492/*TFF2*-308 as A-carrier/CC in this limited study scale (*P *= 1×10^−4^).

**Conclusions:**

The SNPs of *ITGA5*-1160/*ITGB1*-1949/ *ITGB1* + 31804 as T/A/C carriers and *COX-*2-1195/*IL-10*-592 as G-carrier/AA, or more specific to combine *RUNX3* + 492/*TFF2*-308 as A-carrier/CC shall be host factor predisposing to gastric cancer during *H. pylori* infection, and serve as marker to identify high-risk subjects for *H. pylori* eradication.

**Electronic supplementary material:**

The online version of this article (doi:10.1186/s12929-015-0121-7) contains supplementary material, which is available to authorized users.

## Background

*Helicobacter pylori* is defined as a World Health Organization type I carcinogen for gastric cancer [1,2]. *H. pylori*-infected patients may develop gastric cancer after chronic gastritis with gastric atrophy, precancerous lesions such as spasmolytic polypeptide expressing metaplasia (SPEM) or intestinal metaplasia (IM), and dysplasia [3-8]. Gastric cancer typically exhibits familial clustering, and gastric cancer familial relatives (GCF) tend to present with corpus-predominant gastritis and precancerous lesions such as SPEM or IM after *H. pylori* infection [9-13]. Therefore, GCF can be recognized as being at risk of gastric cancer, and such individuals should receive *H. pylori* screening for early bacterial eradication in order to prevent gastric cancer.

The reason why GCF can be at a higher risk of developing gastric cancer after *H. pylori* infection remains uncertain. A more adverse interaction between host and *H. pylori* virulence may exist in GCF to mediate gastric carcinogenesis. The *cagA-vacA-babA2* triple-positive *H. pylori* isolates have been reported as carrying a high risk of gastric cancer, and almost 100% of the *H. pylori* strains are *cagA-vacA-babA2* triple-positive in Taiwan [14-18]. Therefore, GCF in Taiwan are optimal candidates to investigate whether there is any host genomic predisposition in such a high risk group for gastric cancer to determine the specific process of gastric carcinogenesis. In contrast, the duodenal ulcer patients (DU) developed by *H. pylori* infection is well recognized to have a protective host phenotype having a low risk of gastric adenocarcinoma [19]. By examining differences in the selected candidate genes’ single nucleotide polymorphisms (SNPs) between GCF and DU, the study could validate the SNPs potentially correlated to the higher gastric cancer risk in GCF.

This study selected several potential candidate genes for an analysis of SNPs, including those involved in the specific interactions of bacterial virulence and host receptors related with gastric inflammation, precancerous lesions, or even gastric carcinogenesis. We first included the candidate genes *ITGA5* and *ITGB1*, which encode integrin α5β1 gastric epithelial cell surface receptors to adhere with CagL of *H. pylori* to construct injection apparatus to translocate CagA of *H. pylori* via the type IV secretion system (T4SS) [20-22]. As *H. pylori* isolates can directly prime α5β1 integrin of the host, and as the activity of T4SS determines the risk of cancer [20], we investigated whether any host genomic predisposition in *ITGA5* and *ITGB1* existed in high-risk GCF. We also included the interleukin-10 gene (*IL-10*) and cyclooxygenase-2 gene (*COX-2*), both of which participate in chronic inflammation and have been reported to affect gastric cancer outcomes after *H. pylori* infection [16,21-25]. There were two additional SNPs being included, with the runt-related transcription factor 3 gene (*RUNX3*) related to SPEM formation and intestinal-type gastric cancer [26,27], and the trefoil factor 2 (TFF2) gene expressed in SPEM during *H. pylori*-related gastric carcinogenesis [5,28].

The results showed that several candidate genes (including *ITGA5*, *ITGB1*, *IL-10*, *COX-2*, *RUNX3* and *TFF2*) differed in the specific genotypes of SNPs between GCF and DU, and predisposed to pre-cancerous lesion, SPEM, within GCF. These specific genotypes of SNPs may offer candidate markers to screen out those at high risk of gastric cancer for early *H. pylori* eradication in a large-scale study.

## Methods

### Patients and study designs

**Figure 1:**
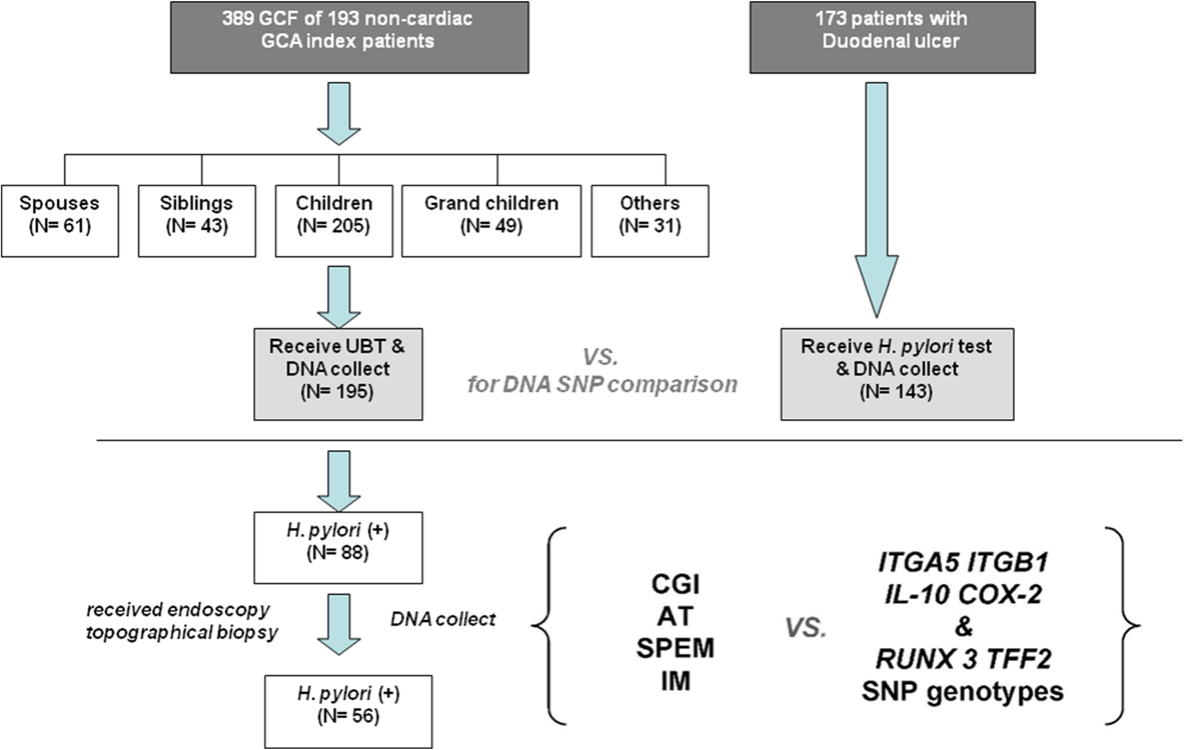
**The study flow chart of case recruitment to screen** ***H. pylori*** **infection.** In total, 389 family relatives (GCF) from 195 non-cardiac gastric cancer (GCA) index patients, including different familial relations, were enrolled. Blood sampling for DNA extraction and *H. pylori* screening with the urea breath test (UBT) were performed in all 389 GCF. There were also 143 of 173 duodenal ulcer patients to receive blood sampling and *H. pylori* screening. The differences in single nucleotide polymorphisms (SNPs) were compared between the DNA of 195 GCA children and 143 duodenal ulcer controls. Of the 88 *H. pylori*-infected children of GCA, 56 received endoscopy and topographical biopsies to assess whether the corpus predominant gastritis index (CGI), atrophy (AT), spasmolytic polypeptide expressing metaplasia (SPEM) and intestinal metaplasia (IM) correlated to the SNPs of *ITGA5*, *ITGB1*, *IL-10*, *COX-2*, *RUNX3*, and *TFF2* identified from the comparison between the children of GCA and the duodenal ulcer controls.

This study prospectively and consequently enrolled 389 family relatives of 193 non-cardiac gastric adenocarcinoma (GCA) index patients and 173 benign DU, all of whom joined a screening project for *H. pylori* infection (NHRI-EX101-9908 BI). A number of the subjects have been referred from the recently published study by our group [12], and the emerging increase of the additional subjects were collected from the funding of the same multi-component grant to fulfill the statistical power required to answer the question whether or not any host genomic predisposition in several candidate genes can exist in the high-risk gastric cancer relatives different to low-risk gastric cancer patients, and to correlate with the presence of precancerous lesions with an increased risk of gastric cancer, especially after *H. pylori* infection. All of the enrolled study subjects provided written informed consent before entering the study. Both the consent form and study design were reviewed by the Research Ethics Committee of National Cheng Kung University Hospital (certification code: HR-98-023). A flow chart of patient enrollment and the familial relationships to the GCA index patients are shown in Figure 1. Among the 389 GCF, there were 61 spouses, 43 siblings, 205 children, 49 grandchildren and 31 other relatives. Each of the enrolled subjects provided blood samples to collect DNA.

The study at first selected the DNA of 195 children of GCA and 143 DU controls to validate whether there were any differences between these two groups with regards to the predisposing risk alleles of SNPs in the *ITGA5, ITGB1, IL-10, COX-2*, *RUNX3*, and *TFF2* genes. All of the 195 children of GCA were also screened for *H. pylori* infection by the ^13^C-urea breath test. For DU, the presence of *H. pylori* infection was confirmed by positive histology results.

Of the 88 *H. pylori*-infected children of GCA with a positive ^13^C-urea breath test, 56 were invited to receive gastric endoscopy to provide topographic gastric biopsy specimens for histological assessments including the corpus predominant gastritis index (CGI), atrophy, SPEM, and IM as described in our previous studies [12,17,29,30]. We further analyzed whether any SNPs associated with specific histology features including CGI, atrophy, SPEM and IM in the children of GCA.

### Blood sampling and SNP identification

The genomic DNA of the participants was extracted from peripheral blood mononuclear cells by the manufacturer’s instructions (Viogene, Taipei, Taiwan). The genes of interest were divided into three groups: 1. integrin α5β1 encoding genes *ITGA5* and *ITGB1*; 2. *IL10* and *COX-2* genes that participate in the inflammation process; and 3. *RUNX3*, and *TFF2* genes relating to late carcinogenesis processes and metaplasia. Because no reports about the relations of diseases for SNPs in *ITGA5*, *ITGB1*, and *TFF2* genes in previous literatures and their allele frequencies in our population, we performed direct sequencing of the promoters of *ITGA5*, *ITGB1*, and *TFF2* genes in 20 randomly-selected GCA offspring and found the candidate SNPs for our study. The other candidate SNPs were identified based on those reported in previous papers, tag SNPs, and those located on the promoter, exon or 3’UTR. We assessed the candidate SNPs with two-step polymerase chain reactions (PCR) and direct sequencing or restriction fragment length polymorphism (RFLP), real time PCR or MassARRAY iPLEX Gold-SNP genotyping. The detailed methods of the identification for 24 candidate SNPs were listed in Additional file 1: Table S1, including *ITGA5* (−1160 G/T;–240 C/T), *ITGB1* (−1949 A/G;–1840 C/T;–1660 -/TTTAA;–1575 A/G;–685-/C; +31804 C/G/T; +32492 A/G), *IL-10* (−1082 A/G;–819 C/T;–592 A/C), *COX-2* (−1195 A/G; +8473 C/T), *RUNX3* (−18508 C/G;–1714 A/C;–1582 C/T;–1166 C/T; +492 A/T; + 73375 A/C) and *TFF-2* (−1373 C/T;–503 A/G;–308 A/C; +4649 A/G).

As in our previous study [31,32], we performed two-step PCR with the following cycling conditions: 94°C for 5 minutes then 15 cycles at 94°C for 30 s, 66°C for 30 s, and 72°C for 60 s; then 25 cycles at 94°C for 30 s, 61°C for 30 s, and 72°C for 60 s, and final elongation at 72°C for 10 minutes. The amplified products were sent for direct sequencing (Genomics BioSci & Tech, Taipei, Taiwan, with an ABI3730XL system) or RFLP with specific restriction enzymes (New England Biolabs, Inc., MA, United States). Some SNPs were identified with real-time PCR. The real-time PCR in this study were performed with TaqMan analysis (PE Applied Biosystems, CA, USA), and the primers were designed by File Builder software. PCR were performed in a 10-μl volume containing 10 ng DNA, 2× Master Mix 5 μl, and 0.5 μl probes. The amplification reaction profile included heating at 95°C for 10 minutes followed by 45 cycles at 95°C for 15 s and 60°C for 60 s. The signal was acquired at 60°C during each cycle. The genotypes were analyzed by SDS 21 software.

We also used a MassARRAY iPLEX Gold-SNP genotyping system to perform genotyping in addition to real-time PCR, PCR-RFLP and direct sequencing. Genotyping with the iPLEX assay involved the assay design, DNA isolation, PCR amplification, clean resin, dispensing to a SpectroCHIP bioarray and matrix-assisted laser desorption ionization time-of-flight mass spectrometry (MALDI-TOF MS) analysis [33]. The assays were performed by Feng Chi Biotech Corp. Taipei, Taiwan, with a Sequenom MassARRAY system and analyzed by the Taipei Institute of Pathology.

### Endoscopy and topographic gastric histology grading

The GCA children with *H. pylori* infection proven by a positive urea breath test were invited to receive endoscopy and topographical biopsies for five specimens from the antrum (*x*2), corpus (*x*2) and high corpus near to the cardia (×1) to assess the related gastric histological features [12,34-37]. Each specimen of the topographic site was reviewed in a blinded manner by the pathologist, and the presence of IM (absence: 0; presence: score 1–3) and atrophy (absence: 0; presence: score 1–3) was assessed according to the modified updated Sydney system [38]. In this study, the presence of IM was strictly defined as at least one gastric biopsy sample showing metaplastic cells (goblet cells) involving at least 5% of the upper third of the gastric mucosa [35-37]. Based on the acute and chronic inflammation scores according to the modified updated Sydney system, the presence of CGI was defined as the occurrence of either: 1) the combination of acute and chronic inflammation scores in the high corpus being equal to or larger than the antrum, but not equal to 1; or 2) the combination of scores in the body being larger than the antrum. All of the other conditions were defined as an absence of CGI, including the combination of acute inflammation score and chronic inflammation score of the antrum and high corpus being equal to 1 [12].

### TFF2 immunohistochemistry for SPEM

We performed TFF2 immunohistochemistry to assess SPEM in the topographical tissues from the antrum, corpus, and high corpus of the *H. pylori*-infected children of non-cardiac gastric cancer patients. Immunohistochemical staining was performed using mouse anti-human spasmolytic polypeptide antibody (GE16C, Leica Biosystem Newcastle Ltd., UK) and a commercial kit (NovoLink™ Polymer Detection System, Leica Biosystem Newcastle Ltd., UK). The protocol was modified from the instructions of the manufacturer as used in our previous study [17]. The presence of SPEM was defined as glands formed by TFF2-expressing cells detected in the specimens of either the corpus or high corpus as described previously [12],17]. Because a whole layer (including the upper, middle and lower portion of the gland) of gastric mucosa is needed to assess SPEM. Any cases where SPEM could not be evaluated were treated as missing data [17]. Thus there were fewer case numbers for the comparison of SPEM.

### Statistics

The Student’s *t* test was used when appropriate to determine parametric differences such as age. We verified the non-parametric proportions such as genomic frequencies of specific SNPs in the GCF and DU patients using mixed effect model with SAS PROC MIXED procedure because the genomic frequencies of the GCF were familial dependent while most DU patients had independent data. To correct multiple testing, permutation test by SAS PROC MULTTEST procedure (20000–50000 permutations) was used. We generated the odds ratios (OR) and 95% confidence intervals (95% CI) between the GCF and DU patients by logistic regression with the adjustment of age and *H. pylori* infection. The linear-by-linear association was applied to assess the frequencies change of the risk SNPs in different familial relation groups. The analyses of the Student’s *t* test, the logistic regression, and the linear-by-linear association were performed using SPSS version 17.0 for Windows (SPSS Inc., Chicago, USA). The analyses of the mixed effect model and permutation test were performed using SAS software version 9.3 (SAS Institute, Cary NC). All tests were 2-tailed with the significance set at a *P* value of less than 0.05.

## Results

### The differences in the selected SNPs between children of GCA and DU

In Figure 1, 195 children of GCA have provided DNA to validate the differences in the SNPs of the *ITGA5, ITGB1, IL-10, COX-2, RUNX3,* and *TFF2* genes, as compared to 143 DU controls. There was no difference in gender between the 195 children of GCA and the 143 DU controls. However, children of GCA had a younger mean age and a lower prevalence of *H. pylori* infection than DU (Table 1). The children of GCA had a higher allele frequencies of *ITGA5*-1160 T-carrier (79% *vs.* 65%; *P *= 0.006), *ITGB*-1949 A-carrier (84% *vs.* 74%; *P *= 0.047), *ITGB1* + 31804 C-carrier (95% *vs.* 88%; *P *= 0.013), *IL-10*-819 TT (52% *vs.* 38%; *P *= 0.014), *IL-10*-592 AA (52% *vs.* 38%; *P *= 0.014) and *COX-2-*1195 G-carrier (83% *vs.* 71%; *P *= 0.019), as compared with DU controls. However, there were no differences in the allele frequencies of the SNPs in the *RUNX3* and *TFF2* genes between the children of GCA and DU controls (Table 1).

**Table 1:** **The differences of the single nucleotide polymorphisms in the** ***ITGA5*** **,** ***ITGB1*** **,** ***IL10*** **,** ***COX-2*** **,** ***RUNX3*** **, and** ***TFF2*** **genes between 195 children of gastric cancer patients and 143 duodenal ulcer patients**

**Parameters N (%)**	**GCA children**	**DU patients**	***P*** *	***P*** **	**OR (95% CI)**
Age (yr)	38.2 ± 10.6	49.2 ± 13.8	1×10^-4^		
Gender (F: M)	99 (51): 96 (49)	59 (41): 84 (59)	0.050	0.096	1.5 (1.0 - 2.3)
*H. pylori* (+: -)	88 (48): 97 (52)	110 (79): 29 (21)	<1×10^-4^	<1×10^-4^	0.2 (0.1 - 0.4)
*ITGA5*					
*ITGA5* -1160	(N = 195)	(N = 139)			
T-carrier: GG	154 (79): 41 (21)	90 (65): 49 (35)	0.017	0.006	2.2 (1.2 - 4.0)
*ITGA5* -240	(N = 193)	(N = 138)			
CC: CT	190 (98): 3 (2)	136 (99): 2 (1)	0.751	1.000	0.3 (0.0 - 3.1)
*ITGB1*					
*ITGB1* -1949	(N = 189)	(N = 136)			
A-carrier: GG	158 (84): 31 (16)	101 (74): 35 (26)	0.047	0.047	2.8 (1.4 - 5.3)
*ITGB1* -1840	(N = 189)	(N = 136)			
CC: T-carrier	100 (53): 89 (47)	75 (55): 61 (45)	0.872	0.735	0.9 (0.6 - 1.6)
*ITGB1* -1575	(N = 189)	(N = 136)			
AA: G-carrier	10 (5): 179 (95)	15 (11): 121 (89)	0.034	0.059	0.3 (0.1 - 0.8)
*ITGB1* -685	(N = 185)	(N = 126)			
-/-: -/C	26 (14): 159 (86)	13 (10): 113 (90)	0.633	0.386	1.5 (0.7 - 3.4)
*IGB1* -1660	(N = 192)	(N = 138)			
TTTAA/TTTAA: others	165 (86): 27 (14)	117 (85): 21 (15)	0.385	0.877	0.9 (0.5 - 1.7)
*ITGB1* +31804	(N = 189)	(N = 136)			
C-carrier: TT	180 (95): 9 (5)	119 (88): 17 (13)	0.003	0.013	4.7 (1.7 - 13.0)
*ITGB1* +32492	(N = 189)	(N = 136)			
G-carrier: AA	97 (51): 92 (49)	57 (42): 79 (58)	0.022	0.113	1.7 (1.0 - 2.9)
*IL10*					
*IL10* -1082	(N = 190)	(N = 138)			
AA: G-carrier	172 (91): 18 (9)	121 (88): 17 (12)	0.216	0.839	1.7 (0.7 - 3.9)
*IL10* -819	(N = 193)	(N = 138)			
TT: C-carrier	101 (52): 92 (48)	53 (38): 85 (62)	0.022	0.014	2.3 (1.4 - 4.0)
*IL10* -592	(N = 193)	(N = 138)			
AA: C-carrier	101 (52): 92 (48)	53 (38): 85 (62)	0.022	0.014	2.3 (1.4 - 4.0)
*COX-*2					
*COX-*2 -1195	(N = 189)	(N = 129)			
G-carrier: AA	156 (83): 33 (17)	92 (71): 37 (29)	0.064	0.019	1.7 (0.9 - 3.2)
*COX-*2 +8473	(N = 194)	(N = 138)			
TT: C-carrier	134 (69): 60 (31)	84 (61): 54 (39)	0.259	0.129	1.4 (0.9 - 2.3)
*RUNX3*					
*RUNX3* -18508	(N = 186)	(N = 135)			
C-carrier: GG	130 (70): 56 (30)	100 (74): 35 (26)	0.586	0.449	0.7 (0.4 - 1.2)
*RUNX3* -1714	(N = 193)	(N = 137)			
CC: A-carrier	109 (56): 84 (44)	68 (50): 69 (50)	0.302	0.261	1.3 (0.7 - 2.1)
*RUNX3* -1582	(N = 189)	(N = 136)			
T-carrier: CC	84 (44): 105 (56)	69 (51): 67 (49)	0.338	0.307	0.9 (0.5 - 1.4)
*RUNX3* -1166	(N = 193)	(N = 137)			
TT: C-carrier	109 (56): 84 (44)	68 (50): 69 (50)	0.302	0.258	1.3 (0.7 - 2.1)
*RUNX3* +492	(N = 189)	(N = 136)			
TT: A-carrier	106 (56): 83 (44)	67 (49): 69 (51)	0.312	0.263	1.2 (0.7 - 2.1)
*RUNX3* +73375	(N = 189)	(N = 135)			
CC: A-carrier	45 (24): 144 (76)	23 (17): 112 (83)	0.122	0.167	1.5 (0.8 - 2.9)
*TFF2*					
*TFF2* -1373	(N = 189)	(N = 136)			
CC: T-carrier	139 (74): 50 (26)	97 (71): 39 (29)	0.692	0.702	1.0 (0.6 - 1.8)
*TFF2* -503	(N = 189)	(N = 136)			
AA: G-carrier	139 (74): 50 (26)	97 (71): 39 (29)	0.692	0.706	1.0 (0.6 - 1.8)
*TFF2* -308	(N = 189)	(N = 136)			
CC: A-carrier	85 (45): 104 (55)	53 (39): 83 (61)	0.394	0.311	1.2 (0.7 - 2.1)
*TFF2* +4649	(N = 188)	(N = 136)			
GG: A-carrier	100 (53): 88 (47)	64 (47): 72 (53)	0.343	0.308	1.4 (0.8 - 2.4)

GCA: gastric cancer. DU: duodenal ulcer. SNPs: single nucleotide polymorphisms. *P value by mixed effect model. **P value by permutation test.

### Combined predisposing SNPs to differentiate between GCA children and DU

Based on the *P* values of permutation tests, we choose SNPs in each gene most significantly different between the case and control groups to derive combined genotypes for further analysis. The study tested the differences of the combined genotypes of the predisposing SNPs in integrin α5β1 encoding genes *ITGA5*, *ITGB1* -1949 and *ITGB1* +31804, implicating the influence of integrin α5β1 on host-bacterial interaction processes during gastric carcinogenesis, between the children of GCA and DU controls (Table 2). The children of GCA had a 5-fold higher risk to have all of the three risk SNPs (*ITGA5* -1160 T-carrier, *ITGB1* -1949 A-carrier, and *ITGB1* +31804 C-carrier), as compared with DU (67% *vs.* 47%; *P* = 0.05). Because of the linkage of disequilibrium between *IL-10* -819 and *IL-10* -592, this study selected *IL-10* -592 to combine with *COX-2* -1195 to indicate a possibly more severe gastric inflammation. This study illustrates that the children of GCA exerted a 3.3-fold higher risk to harbor a combined SNPs in *IL-10*-592/ *COX-2*-1195 as AA/G-carrier than DU controls (43% *vs.* 25%; *P *= 0.005) (Table 2).

**Table 2:** **The comparisons of the genomic difference in the combinations of** ***ITGA5*** **-1160 T-carrier,** ***ITGB1*** **-1949 A-carrier,** ***ITGB1*** **+31804 C-carrier,** ***IL10*** **-592 AA and** ***COX-2*** **-1195 G-carrier between the children of gastric cancer patients and the duodenal ulcer controls**

**Predisposing SNPs**	**Risk SNPs N (%)**	**GCA children**	**DU controls**	***P*** *	***P*** **	**OR (95% CI)**
*ITGA5* -1160		(N = 189)	(N = 134)			
T-carrier	3R:2R:1R:0R	126(67): 49(26): 12(6): 2(1)	63(47): 48(36): 18(13): 5(4)	0.002		
*ITGB1* -1949						
A-carrier	3R	126 (67)	63 (47)	0.225	0.050	5 (0.9 - 26.5)
*ITGB1* +31804	2R	49 (26)	48 (36)	0.539	0.514	2.6 (0.5 - 13.8)
C-carrier	1R	12 (6)	18 (23)	0.575	0.756	1.7 (0.3 - 10.0)
	0R	2 (21)	5 (4)			1
*COX-*2 -1195		(N = 188)	(N = 125)			
G-carrier	2R: 1R: 0R	80 (43): 93 (49): 15 (8)	31 (25): 75 (60): 19 (15)	0.013		
With or without						
*IL-10* -592 AA	2R	80 (43)	31 (25)	0.021	0.005	3.3 (1.5 - 7.2)
	1R	93 (49)	75 (60)	0.457	0.344	1.6 (0.7 - 3.3)
	0R	15 (8)	19 (15)			1
*ITGA5*-1160		(N = 185)	(N = 124)			
T-carrier	2R: 1R: 0R	44 (24): 115 (62): 26 (14)	10 (8): 67 (54): 47 (38)	<1×10^-4^		
*ITGB1* -1949						
A-carrier						
*ITGB1* +31804	2R	44 (24)	10 (8)	<1×10^-4^	<1×10^-4^	8.0 (3.4 - 18.4)
C-carrier	1R	115 (62)	67 (54)	2×10^-4^	3×10^-4^	3.1 (1.8 - 5.5)
With or without	0R	26 (14)	47 (38)			1
*COX-2* -1195						
G-carrier						
*IL10* -592 AA						
		(N = 193)	(N = 135)			
*IL-10* -592 AA	2R: 1R: 0R	74 (38): 105 (54): 14 (7)	33 (24): 73 (54): 29 (21)	4×10^-4^		
With or without						
*ITGA5* -1160	2R	74 (38)	33 (24)	1×10^-4^	<1×10^-4^	4.6 (2.2 - 9.9)
T-carrier	1R	105 (54)	73 (54)	6×10^-4^	0.003	3.0 (1.5 - 6.0)
	0R	14 (7)	29 (21)			1

GCA: gastric cancer. DU: duodenal ulcer. SNPs: single nucleotide polymorphisms. 3R: the presence of 3 predisposing SNPs with risk alleles. 2R: the presence of 2 predisposing SNPs with risky alleles.

1R: the presence of 1 predisposing SNP with risk allele. 0R: no any predisposing SNPs with risk alleles. *P value by mixed effect model; **P value by permutation test.

We further combined the above two sets to increase the relative risk to differentiate the children of GCA children to DU. Also in Table 2, the children of GCA had a 8.0-fold higher risk (*P* < 1×10^−4^) to have a combined genotype with *ITGA5*-1160/*ITGB1*-1949/ *ITGB1* + 31804 as T/A/C carriers and *COX-*2-1195/*IL-10*-592 as G-carrier/AA than DU controls. To simplify the combination, we selected the SNPs most significantly different allele frequencies between GCA children and DU controls, *ITGA5* -1160 T-carrier and *IL-10* -592 AA, to serve as risk genotypes. The children of GCA had a 4.6-fold risk of having both risk genotypes as compared to DU controls (*P* < 1×10^−4^).

### The combined SNPs correlate to SPEM in children of GCA

In Figure 1, 56 children of GCA with *H. pylori* infection received endoscopy to assess the histology features. The study illustrated the combined genotype with *ITGA5*-1160/*ITGB1*-1949/*ITGB1* + 31804 as T/A/C carriers and *COX-*2-1195/*IL-10*-592 as G-carrier/AA correlated to the presence of SPEM (*P *= 0.016), but not CGI, atrophy or IM (*P* > 0.05). The children of GCA with the combined genotype with *ITGA5*-1160/*ITGB1*-1949/*ITGB1* + 31804 as T/A/C carriers and *COX-*2-1195/*IL-10*-592 as G-carrier/AA had a 5.25-fold higher risk to have SPEM (*P *= 0.016) (Table 3). Although the children of GCA had a 4.6-fold higher chance of being *ITGA5*-1160/*IL-10*-592 as T-carrier/AA, but such a simplified combined genotype did not correlate to CGI, atrophy, SPEM or IM (*P* > 0.05).

**Table 3:** **The difference of the H. pylori-related histological features between the children of gastric cancer patients with and without the risk allele genotypes as ITGA5-1160 T-carrier/ITGB1-1949 A-carrier/ITGB1+31804 C-carrier & COX-2-1195 G-carrier/IL10-592 AA**

**Risks N (%) Pathology**	***ITGA5*** **-1160/** ***ITGB1*** **-1949/** ***ITGB1*** **+31804/** ***COX-2*** **-1195/** ***IL10*** -592 as T*/A*/C*/G*/AA	**Others**	**P** *	**P** **	**OR (95% CI)**
CGI (N = 56)					
Present: absent	14 (58): 10 (42)	15 (47): 17 (53)	0.115	0.429	1.6 (0.5-4.6)
Atrophy (N = 56)					
Present: absent	17 (71): 7 (29)	21 (66): 11 (34)	0.932	0.778	1.3 (0.4-4.0)
SPEM (N = 48)					
Present: absent	18 (82): 4 (18)	12 (46): 14 (54)	0.010	0.016	5.3 (1.4-19.8)
IM (N = 56)					
Present: absent	4 (17): 20 (83)	6 (19): 26 (81)	0.831	1.000	0.9 (0.2-3.5)

CGI: corpus predominant gastritis index. SPEM: spasmolytic polypeptide-expressing metaplasia. IM: intestinal metaplasia. *Indicated the carrier of the specific allele in SNPs. *P value by mixed effect model; **P value by permutation test.

Since *RUNX3* and *TFF2* regulate the SPEM formation during gastric carcinogenesis [26-28], we further tested if any specific SNPs in *RUNX3* and *TFF2* genes correlated to the presence of SPEM in the children of GCA. In Table 4, the children of GCA with SPEM had a higher allele frequencies of *RUNX3* + 492 as A-carrier (53% *vs.* 22%; *P *= 0.042) and *TFF2*-308 as CC (60% *vs.* 28%; *P *= 0.041) than those without SPEM. Thus we further analyzed the combined genotypes of these 2 SNPs. Nearly 90% of the children of GCA with SPEM were either *RUNX3* + 492 A-carrier or *TFF2*-308 CC and these two genotypes led to a 11.3-fold increased risk of having SPEM in the children of GCA (*P *= 0.001) (Table 4). However, there was no significant correlation between the *ITGA5*-1160/*ITGB1*-1949/*ITGB1* + 31804/*COX-*2-1195/*IL-10*-592 as T-carrier/A-carrier/C-carrier/G-carriers/AA and the *RUNX3* + 492/ *TFF2*-308 as A-carrier/CC among the children of GCA. By combining these two sets of SNPs, the risk of getting SPEM within children of GCA elevated up to 112 folds (*P* < 1×10^−4^) (Table 5).

**Table 4:** **The differences of the single nucleotide polymorphisms in the** ***RUNX3*** **, and** ***TFF2*** **genes between children of gastric cancer patients with (N = 31) or without (N = 18) SPEM**

**Parameters N (%)**	**SPEM (+)**	**SPEM (-)**	***P*** *	***P*** **	**OR (95% CI)**
Age (yr)	39.9 ± 7.7	38.2 ± 11.3	0.312		
Gender (F: M)	20 (65): 11 (35)	7 (39): 11 (61)	0.092	0.136	2.9 (0.9 - 9.5)
*RUNX3*					
*RUNX3* -18508	(N = 29)	(N = 17)			
C-carrier: GG	21 (72): 8 (28)	12 (71): 5 (29)	0.897	1.000	1.1 (0.3 - 4.1)
*RUNX3* -1714	(N = 31)	(N = 18)			
A-carrier: CC	16 (52): 15 (48)	4 (22): 14 (78)	0.044	0.067	3.7 (1.0 - 13.9)
*RUNX3* -1582	(N = 30)	(N = 18)			
T-carrier: CC	16 (53): 14 (47)	5 (28): 13 (72)	0.085	0.137	3.0 (0.8 - 10.4)
*RUNX* -1166	(N = 31)	(N = 18)			
C-carrier: TT	16 (52): 15 (48)	4 (22): 14 (78)	0.044	0.070	3.7 (1.0 - 13.9)
*RUNX3* +492	(N = 30)	(N = 18)			
A-carrier: TT	16 (53): 14 (47)	4 (22): 14 (78)	0.035	0.042	4.0 (1.1 - 15.0)
*RUNX3* +73375	(N = 30)	(N = 18)			
C-carrier: AA	21 (70): 9 (30)	14 (78): 4 (22)	0.613	0.741	0.7 (0.2 - 2.6)
*TFF2*					
*TFF2* -1373	(N = 30)	(N = 18)			
C-carrier: TT	30 (100): 0 (0)	17 (94): 1 (6)	0.206	0.377	2.6 (0.8 - 9.2)
*TFF2* -503	(N = 30)	(N = 18)			
AA: G-carrier	23 (77): 7 (23)	10 (56): 8 (44)	0.127	0.194	2.6 (0.7 - 9.2)
*TFF2* -308	(N = 30)	(N = 18)			
CC: A-carrier	18 (60): 12 (40)	5 (28): 13 (72)	0.022	0.041	3.9 (1.1-13.8)
*TFF2* +4649	(N = 30)	(N = 18)			
G-carrier: AA	26 (87): 4 (13)	17 (94): 1 (6)	0.419	0.637	0.4 (0.1-3.7)
*RUNX3+492/TFF2-308*					
A-carrier/CC					
2R	7 (23)	1 (6)	0.001	0.009	23.3 (2.0 - 273.3)
1R	20 (67)	7 (39)	0.001	0.004	9.5 (2.0 - 44.9)
0R	3 (10)	10 (56)			1
2R+1R:0R	27 (90): 3 (10)	8 (44): 10 (56)	3×10^-4^	0.001	11.3 (2.5 - 51.0)

SPEM: Spasmolytic polypeptide-expressing metaplasia; SNPs: single nucleotide polymorphisms. 2R: the presence of 2 predisposing SNPs with risk alleles. 1R: the presence of 1 predisposing SNP with risk allele. 0R: no any predisposing SNPs with risk alleles. *P value by mixed effect model; **P value by permutation test.

**Table 5:** **The risk of SPEM increases stepwise for** ***ITGA5*** **-1160 T-carrier,** ***ITGB1*** **-1949 A-carrier,** ***ITGB1*** **+31804 C-carrier,** ***COX-2*** **-1195 G-carrier, and** ***IL10*** **-592 AA to combine with** ***RUNX3*** **+492 as A-carrier** & ***TFF2*** **-308 as CC in the children of gastric cancer patients**

**Predisposing SNPs**	**Risk SNPs N (%)**	**SPEM (+)**	**SPEM (−)**	***P*** *	***P*** **	**OR (95% CI)**
*ITGA5*-1160/*ITGB1*-	2R: 1R: 0R	16 (53): 13 (43): 1 (3)	1 (6): 10 (56): 7 (39)	<1×10^−4^		
1949/*ITGB1*+31804/*COX-2*-1195/*IL10*-592	2R: 1R+ 0R	16 (53): 14 (47)	1 (6): 17 (94)	5×10^4^	0.001	19.4 (2.3-165.2)
as T*/A*/C*/G*/AA						
	2R+ 1R: 0R	29 (97): 1 (3)	11 (61): 7 (39)	5×10^−4^	0.003	18.5 (2.0-167.8)
with or without						
	2R	16 (53)	1 (6)	1×10^−4^	1×10^−4^	112.0 (6.1-2057.3)
*RUNX3*+492/*TFF2*-308	1R	13 (43)	10 (56)	0.045	0.0518	9.1 (1.0-86.5)
as A*/CC	0R	1 (3)	7 (39)			1

SPEM: spasmolytic polypeptide-expressing metaplasia. SNPs: single nucleotide polymorphisms. 2R: the presence of 2 predisposing SNPs with risk alleles. 1R: the presence of 1 predisposing SNPs with risk allele. 0R: no any predisposing SNPs with risk alleles. *Indicated the carrier of the specific allele in SNPs. *P value by mixed effect model; **P value by permutation test.

### Combined SNPs predisposed to SPEM among different familial relative groups of GCA

**Figure 2:**
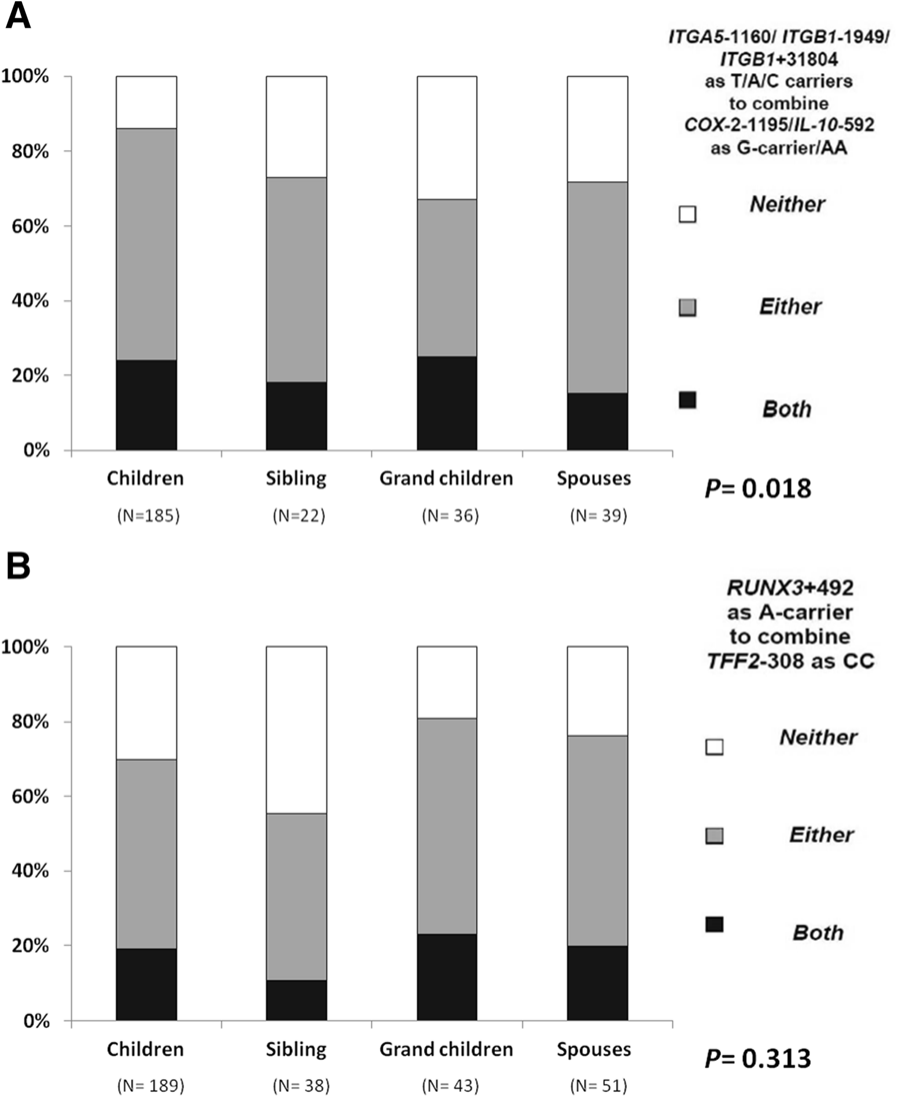
**The combined risk genotypes’ distribution in gastric cancer familial relatives. A**. The prevalences of the *ITGA5*-1160/ *ITGB1*-1949/*ITGB*1 + 31804 as T-carrier/A-carrier/C-carrier and *COX*-2-1195/*IL10*-592 as G-carrier/AA among different GCA familial relative groups were shown. The children of gastric cancer patients had higher prevalence of the presence of either or both risk combination SNPs in *ITGA5*-1160/*ITGB1*-1949/*ITGB1* + 31804 as T/A/C carriers and *COX-2*-1195/*IL-10*-592 as G-carrier/AA than other familial relative groups (*P* = 0.018). **B**. There were no difference in the combined genotype of *RUNX3* + 492/*TFF2*-308 as A-carrier/cm^3^ among different familial groups of gastric cancer patients (*P *= 0.313).

We further assessed whether the different gastric cancer familial relative groups had different proportions of the combined SNPs predisposing to SPEM. In Figure 2A, the offspring, including children and grandchildren, of GCA had a higher rate of *ITGA5*-1160/*ITGB1*-1949/*ITGB1* + 31804/*COX-*2-1195/*IL-10*-592 as T-carrier/A-carrier/C-carrier/G-carriers/AA than other familial groups (*P *= 0.018). However, in Figure 2B, there was no difference in the combined genotype of *RUNX3 + 492/TFF2-308* as A-carrier/CC among the different familial groups of GCA (*P *= 0.313). Furthermore, children of the GCA index patients carrying the combination sets of *ITGA5*-1160/*ITGB1*-1949/*ITGB1* + 31804/*COX-*2-1195/*IL-10*-592 as T-carrier/A-carrier/C-carrier/G-carriers/AA also had a higher prevalence of the same combination SNPs than children of GCA index patients not carrying such combinations of SNPs (43% *vs.* 16%; *P *= 0.003; OR 3.9; 95% CI: 1.6-9.6).

## Discussion

There were genomic predispositions of SNPs in the *ITGA5*, *ITGB1*, *IL-10* and *COX-2* genes in the children of GCA, who are considered to be a risk group of gastric cancer after *H. pylori* infection. The combined genotype as *ITGA5*-1160/*ITGB1*-1949/*ITGB1* + 31804 as T/A/C carriers and *COX-*2-1195/*IL-10*-592 as G-carrier/AA significantly predisposed to the precancerous lesions such as SPEM after *H. pylori* infection in children of GCA. Such a predisposing risk of SPEM can be elevated via combination with *RUNX3* + 492/*TFF2*-308 as A-carrier/ CC in children of GCA. The combined genotype such as *ITGA5*-1160/*ITGB1*-1949/*ITGB1* + 31804 as T/A/C carriers and *COX-*2-1195/*IL-10*-592 as G-carrier/AA, or even more specifically combined with *RUNX3* + 492/*TFF2*-308, may thus serve as a host factor to identify the risk group of gastric cancer for an early *H. pylori* eradication.

The study utilized patients with duodenal ulcers to serve as cohort controls with the least gastric cancer risk. In a review of the related literature, gastric cancer relatives have been recognized as having an increased gastric cancer risk. Gastric cancer relatives exist with a genomic predisposition to gastric cancers, especially after *H. pylori* infection. The study thus attempted to illustrate whether the possible candidate genomic polymorphisms selected in this study can be different between our high-risk gastric cancer relatives and low-risk duodenal ulcer patients in either Tables 1 and 2. In this study, the children of GCA had a younger mean age than DU patients, whose mean age was compatible to that of DU in a previous study [19]. Thus, our enrolled DU patients were found to be suitable to serve as the low-cancer risk comparison group.

The activity of T4SS mediated by *H. pylori* positively associated with the risk of gastric cancer [20]. Furthermore, we disclosed that *H. pylori* isolates with the *cagL* amino acid sequence polymorphism Y58E59 could exploit higher amounts of gastric integrin α5β1 to mediate stronger T4SS activity [39,40]. Namely, more toxic *H. pylori* isolates shall have mediated with higher gastric cancer risk via the host integrin α5β1 expressions. The current study showed that the children of GCA, who are at a high risk of gastric cancer, can express with a 5-fold higher risk to be *ITGA5*-1160/*ITGB1*-1949/*ITGB1* + 31804 as T/A/C carriers in *ITGA5* and *ITGB1* than DU (Table 2). This suggests that there should be a host predisposition to regulate the expression of the putative integrin α5β1 in addition to the virulence factor of *H. pylori* isolates. Thus, it would be worthwhile to validate the correlation of *ITGA5* and *ITGB1* genes to the *H. pylori-*related histological precancerous changes, especially among children of GCA.

The current study also identified that the children of GCA patients as having a higher prevalence of *IL-10*-819 as TT and *IL-10*-592 as AA than DU, compatible with previous findings to show such genotypes with lower IL-10 excretion and more severe gastritis [21-23,41]. COX-2 over-expression can be evident in pre-malignant gastric lesions after *H. pylori* infection [11,24,25]. Our data showed that the children of GCA had a higher prevalence of *COX-2-*1195 G-carrier than DU, but the finding is not compatible with a previous study, which suggests that the *COX-2-*1195 AA genotype carries a higher risk of GCA [42]. This may also be due to potential ethnic differences, or that the *COX-2-*1195 G-carrier may just serve to associate with other pathways of gastric carcinogenesis [43].

Because gastric carcinogenesis is a multi-step and multi-factorial process, we anticipate that SNPs on different genes work in concert in the gastric carcinogenesis. We choose TagSNPs in each gene as the most significantly different between the GCF and the DU patients for the analysis of combined genotypes. We found that the children of GCA had an 8-fold risk to have the combined genotypes of either a complicated model (*ITGA5*-1160/*ITGB1*-1949/*ITGB1* + 31804 as T/A/C carriers and *COX-*2-1195/*IL-10*-592 as G-carrier/AA) (Table 2), higher than the risk difference of each single SNP. This suggests that when more risk alleles combine, the higher the risk of gastric cancer. Nevertheless, only the first complicated model can be related to the presence of the precancerous lesions as SPEM in the children of GCA (Table 3). It thus suggested the complicated model with 5 risk alleles shall be a novel host factor to determine the high-risk patients of gastric cancer for early *H. pylori* eradication.

The combined genotypes *ITGA5*-1160/*ITGB1*-1949/*ITGB1* + 31804 as T/A/C carriers and *COX-*2-1195/*IL-10*-592 as G-carrier/AA can just correlate with SPEM, but not IM. The lack of correlation to IM may be due to the small number of cases with IM or the younger mean age in our series, or to fulfill SPEM is generally earlier than IM [6,44,45].

In Table 4, the combined genotype of *RUNX3* + 492/*TFF2*-308 as A-carrier/cm^3^ was also correlated to the presence of SPEM in the children of GCA. Accordingly, combining these 2 sets of risk combined genotypes, the risk to get SPEM increased up to 112 folds (Table 5). This indicated these 2 sets of combined genotypes can be synergistic to enhance the SPEM. With regards to no significant difference of allele frequencies of SNPs in *RUNX3* and *TFF2* genes between the children of GCA with low risk DU, the host-bacterial interaction regulated by integrin α5β1 and the chronic inflammation regulated by IL-10 and COX-2 may be prior to the regulation of RUNX3 and TFF2 in the gastric carcinogenesis process.

The allele frequencies of the combination of *ITGA5*-1160/*ITGB1*-1949/*ITGB1* + 31804 as T-carrier/A-carrier/C-carriers in GCA children was 67%, which was higher than 47% in DU patients (*P = *0.0007). When taking more SNPs with *COX-*2-1195/*IL-10*-592 and *RUNX3*/*TFF2* into consideration, the allele frequencies of the complicated model (*ITGA5*-1160/*ITGB1*-1949/*ITGB1* + 31804 as T/A/C carriers, *COX-*2-1195/*IL-10*-592 as G-carrier/AA, and *RUNX3*/*TFF2* as A-carrier/cm^3^) remained higher in children of GCA than in DU (17% *vs.* 6%, *P *= 0.003). This data suggests the greater number of risk SNPs included, the more specific the result to indicate who the high risk group was.

The study has limitations to consider such as that it does not divide the *H. pylori*-positive and negative offspring of gastric cancer into analysis due to the case scales. Furthermore, another limitation of the study is the small sample size, especially the GCF with available pathology data. This made the significance of the risk combined genotypes to correlate with SPEM limited despite there being a high odds ratio. This was because many GCF were unwilling to receive gastric endoscope examination although we screened 389 GCF and 205 children of non-cardiac GCA patients as outlined in Figure 1. This indicated a lack of insight into the general population, even with GCF having high GCA risks. It also highlights the importance of studying markers to identify high-risk groups of GCA. We do agree if budgeting allows, eradicating *H. pylori* infection for all offspring of gastric cancer patients remains ideal. If we can understand the genomic predispositions from offspring with a higher risk of having an adverse outcome, such host factors may be helpful to screen out the general dyspepsia patients for early *H. pylori* eradication to prevent gastric cancer. The relevance of this study is that it answers the reason why offspring of gastric cancer are at risk, and that it also provides clinicians with a potential tool to help screen out the higher risk groups with obvious cost-benefit concerns.

## Conclusions

There were SNPs in the *ITGA5*, *ITGB1*, *IL-10*, and *COX-2* genes in the children of GCA. The combined genotypes *ITGA5*-1160/*ITGB1*-1949/*ITGB1* + 31804 as T/A/C carriers and *COX-*2-1195/*IL-10*-592 as G-carrier/AA could determine the risk of SPEM after *H. pylori* infection. Such risk can be elevated up to 112-fold by combining *RUNX3* + 492/*TFF2*-308 as A-carrier/cm^3^. The host genomic SNPs shall be novel to identify the high-risk patients of gastric cancer for early *H. pylori* eradication.

## Additional file

Additional file 1: Table S1. The identification number, identifying methods and study methods of the target single nucleotide polymorphisms (SNPs) in this study.

## Abbreviations

AT: Atrophy
CGI: Corpus predominant gastritis index
COX-2: Cycloxygenase-2
DU: Duodenal ulcer patients
GCA: Gastric cancer patients
GCF: Family relatives of gastric cancer patients
IM: Intestina metaplasia
IL-10: Interleukin 10
ITGA: Integrin alpha: ITGB: Integrin beta
RUXN3: Runt-related transcription factor 3
SPEM: Spasmolytic polypeptide-expressing metaplasia
TFF2: Trefoil factor 2
T4SS: Type four secretion system
PCR: Polymerase chain reactions
RFLP: Restriction fragment length polymorphism
