# Supplementary material for: Genomic single nucleotide polymorphisms in the offspring of gastric cancer patients predispose to spasmolytic polypeptide-expressing metaplasia after H. pylori infection
Source: J Biomed Sci. 2015 Feb 21;22(1):16. doi: 10.1186/s12929-015-0121-7 (PMC4340867; doi:10.1186/s12929-015-0121-7)
Supplement: Additional file 1: Table S1. — The identification number, identifying methods and study methods of the target single nucleotide polymorphisms (SNPs) in this study. [file 12929_2015_121_MOESM1_ESM.doc]

**Additional file 1: Table S1. The identification number, identifying methods and study methods of the target single nucleotide polymorphisms (SNPs) in this study**

| **Gene** | **SNPs** | **SNPs ID** | **Location** | **Identifying method** | **Study method** |
| --- | --- | --- | --- | --- | --- |
| ***ITGA5*** | -1160 G/T  -240 C/T | rs2091768  rs75795923 | promoter  promoter | direct sequencing  direct sequencing | real-time PCR  real-time PCR |
| ***ITGB1*** | -1949 A/G  -1840 C/T  -1660 -/TTTAA  -1575 A/G  -685 -/C  +32492 A/G  +31804 C/G/T | rs1187079  Novel  rs67479665  rs1187077  rs35016806  rs2298141  rs2230394 | promoter  promoter  promoter  promoter  promoter  exon6  exon5 | direct sequencing  direct sequencing  direct sequencing  direct sequencing  direct sequencing  tag SNP  tag SNP | mass array  mass array  real-time PCR  mass array  mass array  mass array  mass array |
| ***IL10*** | -1082 A/G  -819 C/T  -592 A/C | rs1800896  rs1800871  rs1800872 | promoter  promoter  promoter | paper  paper  paper | direct sequencing  direct sequencing  direct sequencing |
| ***COX2*** | -1195 A/G  +8473 C/T | rs689466  rs5275 | promoter  3’-UTR | paper  tag SNP | RFLP (*Pvu II*)  real-time PCR |
| ***RUNX3*** | -18508 C/G  -1714 A/C  -1582 C/T  -1166 C/T  +492 A/T  +73375 A/C | rs423539  rs7414934  rs7536201  rs7528484  rs6672420  rs7519472 | 5’ upstream  distal promoter  distal promoter  distal promoter  exon1  3’downstream | paper  paper  paper  paper  paper  paper | mass array  real-time PCR  mass array  real-time PCR  mass array  mass array |
| ***TFF2*** | -1373 C/T  -503 A/G  -308 A/C  +4649 A/G | rs3814897  rs3814896  rs13052596  rs225334 | promoter  promoter  Promoter  3’-UTR | direct sequencing  direct sequencing  direct sequencing  tag SNP | mass array  mass array  mass array  mass array |
